# Analysis of NAMCS data for multiple sclerosis, 1998–2004

**DOI:** 10.1186/1741-7015-5-6

**Published:** 2007-04-05

**Authors:** Jagannadha R Avasarala, Cormac A O'Donovan, Steve E Roach, Fabian Camacho, Steven R Feldman

**Affiliations:** 1 Kansas Neurological Consultants,* Wichita, KS 67218, USA; 2 Department of Neurology, Wake Forest University School of Medicine, Winston-Salem, NC 27157, USA; 3 Department of Neurology, The Ohio State University Medical Center, Columbus, OH 43210 USA; 4 Department of Dermatology, Wake Forest University School of Medicine, Winston-Salem, NC 27157, USA

## Abstract

**Background:**

To our knowledge, no study to date has investigated the prescribing patterns of immunomodulatory agents (IMAs) in an outpatient setting in the United States. To address this issue, we performed retrospective data analyses on National Ambulatory Medical Care Survey (NAMCS) data for MS patient visits between 1998 and 2004.

**Methods:**

NAMCS data are a weighted estimate of the nationwide frequency of patients' outpatient clinic visits. We analyzed NAMCS data in the following categories: (1) the proportion of MS patient visits to neurologists, family practitioners or internists, (2) age/gender/race/geographical distribution patterns in patient visits, and (3) the proportion of patients on IMA treatment among established MS patients.

**Results:**

There were an estimated 6.7 million multiple sclerosis (MS) patient visits to the clinics between 1998–2004. Neurologists recorded the most patient visits, 50.7%. Patient visits were mostly in the fourth and fifth decade age group (57.9%). The male to female ratio was 1:4. No statistical evidence was observed for a decline or increase in IMA usage. About 62% patients visiting neurologists and 92% seen by family practitioners/internists were not using IMAs. Our results suggest that between the years 1998–2003, the use of interferon-1a tended to decline while the use of interferon-1b and glatiramer acetate, increased.

**Conclusion:**

Strategies that lead to improved use of IMAs in the management of MS in the outpatient setting are needed.

## Background

It is well established that MS is an inflammatory demyelinating disease of the central nervous system that, after trauma, is the second most common cause of neurologic disability in young adults [1,2]. MS is a clinical diagnosis and requires the integration of patient history with neurological examination, radiological evidence [3] and relevant laboratory tests. It is accepted that MS is about twice as common in women than in men [4]. The cause of MS is unknown but environmental factors [5] and multiple gene loci probably contribute to disease onset [6,7].

The course of MS depends in part on the type of disease (relapsing-remitting or primary progressive) and site(s) of lesions. MS is a disease characterized by inter and intra-patient variations, making prognosis difficult. Over time, symptoms tend to become permanent and progressive disability ensues. Since the early 1990s, six IMAs have been approved by the US Federal Drug Administration (FDA) for use in MS therapy: interferon-1b, (Betaseron) in 1993, interferon-1a (Avonex) and glatiramer acetate (GA, Copaxone) in 1996, interferon-1a (Rebif) in 2002, mitoxantrone (Novantrone) in 2000 and Tysabri (Natalizumab) in 2006. Treatment with IMAs is thought to reduce the frequency of relapses and slow disease progression as shown in pivotal studies [8-11]. Economic modeling suggests that treatment with IMAs is probably cost-effective [12]. Although the costs of treatment remain high, loss of productivity and direct care costs for individuals disabled by MS are higher with worsening expanded disability status scores (EDSS). Published data suggest that worsening EDSS from < 3.5 to > 6.5 increase the mean total cost per year of MS by a factor of 4.39 [13]. Therefore, analyses about outpatient management practices could lead to improvement in the treatment of MS patients and design of better cost-effective treatment practices.

## Methods

First initiated in 1973, NAMCS is a national probability sample survey conducted in the United States and collects data on the utilization of ambulatory medical care services provided by office-based physicians. The National Center for Health Statistics (NCHS) and the Centers for Disease Control and Prevention (CDC; [14]) conducts NAMCS and it is particularly designed to meet the need for objective, reliable information about the provision and use of ambulatory medical care services in the United States. Findings are based on a sample of visits to non-federally employed office-based physicians who are primarily engaged in direct patient care. Physicians in the specialties of anesthesiology, pathology, and radiology are excluded from the survey. The survey was conducted annually from 1973 to 1981, then in 1985, and annually again since 1989.

To collect data for NAMCS, specially trained interviewers visit the physicians prior to their participation in the survey in order to provide them with survey materials and instruct them on how to complete the forms. Data collection from the physician, rather than from the patient, provides an analytic base that expands information on ambulatory care collected through other NCHS surveys. Each physician is randomly assigned to a 1-week reporting period. During this period, data for a systematic random sample of visits are recorded by the physician or office staff on an encounter form provided for that purpose. Data are obtained on patients' symptoms, physicians' diagnoses, and medications ordered or provided. The survey also provides statistics on the demographic characteristics of patients and services provided, including information on diagnostic procedures, patient management, and planned future treatment.

The NAMCS survey contains data collected on individual outpatient office visits and is then weighted to reflect national estimates. The NAMCS data can be used to address questions about patterns of reporting disease, disease-specific patient characteristics, and diagnoses. The basic sampling unit for NAMCS is the physician-patient encounter or outpatient visit.

For sample design, NAMCS uses a multi-stage approach comprised of probability samples of primary sample units (PSUs), physician practices within PSUs, and patient visits within practices. Practices studied are those of non-federally employed physicians classified by the American Medical Association (AMA) or American Orthopedic Association (AOA) as "office-based, patient care". In addition, the practicing physicians had to meet four requirements as listed below. Furthermore, the sample design describes that the design used was a multistage probability sampling design, that the sample consisted of 112 PSUs that comprised a probability sub-sample of the PSUs used in the NHIS survey (National Health Interview survey), that the second stage of the design consisted of a probability sample of physicians within each PSU selected from master files in the AMA or AOA, and finally that the third stage involved randomly sampling the visits for a physician using the above sampling rates as determined in a pre-survey interview. The third stage involved two steps: (1) the total physician sample was divided into 52 approximately equal sub-samples that were then randomly assigned to one of the 52 weeks in a survey year; and (2) a systematic random sample of visits was selected by the physician during the assigned week. Patient recording includes two forms, a patient log and a patient record completed by the physician with the assistance of office staff. The patient log is a sequential list of patients seen in the office during the period 1998–2004.

To extrapolate to national estimates, each individual record is assigned an inflation factor called the patient visit weight, which is then used to predict the total number of office visits made in the US. All estimates from the NAMCS are related to the number of patient visits and subject to sampling variability. An estimate is considered reliable if it has a relative SE (sampling errors) of ≤ 30% of the estimate, per NCHS standards. All data management and analyses described were performed using SAS software (Statistical Analysis System; SAS Institute; Cary, NC, USA).

We focused on IMA treatment trends between 1998–2004 for all FDA approved IMA drugs. We chose to analyze data on IMA use only among established MS patients as initial visits to physicians could be part of a diagnostic work-up and IMA use may not be initiated during the first visit. Visits by established patients (return visits) were identified if the patient was classified as having been seen before.

In order to test for the categorical association between various characteristics of the sample with year, chi-square testing for homogeneity of proportions (Wald) was performed. We accomplished this using the SAS 'Surveyfreq' procedure and adjusted for the survey design in the NAMCS, taking into consideration the NAMCS sample weights, stratification, and clustering variables. Table 1 shows temporal trends in sample demographics and in the use of FDA approved agents.

Logistic regression analysis was also performed to compare the adjusted odds of IMA prescription between years, with the dependent variable based on the probability of an IMA prescription in MS return visits. The explanatory variables were year (treated as categorical variable), age group, gender, physician specialty, race, and insurance type. The SAS procedure 'Surveylogistic' was used to calculate odds ratios and survey-corrected confidence intervals. Statistical significance of observed differences was set at p < 0.05. In our study, MS patient visit was defined on the basis of International Statistical Classification of Diseases and Related Health Problems 9 (ICD-9) coding of 340.

## Results

Table 1 shows the demographics for overall MS visits and return visits by physician type. An estimated 6.7 million patient visits (MS) occurred between 1998–2004 at a rate of 3.4 visits/1,000 persons/year. The visit rate in the metropolitan statistical area (MSA) versus non-MSAs was 2.73 to 0.64 per 1,000 persons, respectively. A total of 56% of all MS visits in the MSA were to neurologists compared to 26% in non-MSAs. Women had a higher visit rate (4:1) compared to men, and Caucasians had a higher visit rate compared to African-Americans (90% versus 8%).

Up to 32% of all office visits with an MS diagnosis were estimated to be to the patient's primary care physician. Among visits made to offices in 2003, 52% listed private insurance, followed by Medicare at 27%, and Medicaid at 10.5% (data not shown). Neurologists accounted for 50.7% MS patient visits while general/family practice and internists combined provided for 33.7% of the visits. Patient visits were highest in the West (29%) compared to the Midwest (19.4%). Proportions for return visits to all the three physician groups were very similar to the overall MS visit proportions.

**Figure 1:**
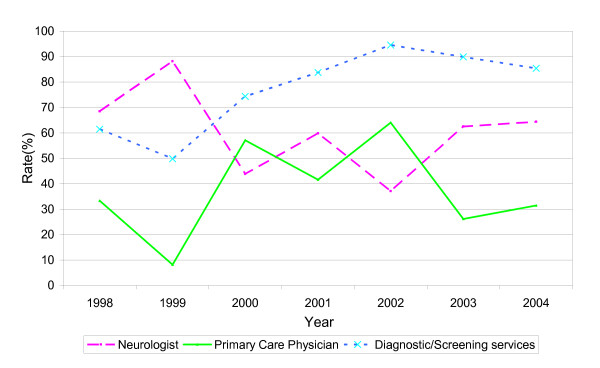
Trends in physician return visits with a diagnosis of MS, NAMCS data, 1998–2004. Y-axis depicts all returning patients with MS diagnosis.

When compared by year, the proportion of visits for primary care physician (p < 0.0001), diagnostic/screen services ordered/provided (p = 0.0182), versus visits to neurologist (p = 0.0079), differed significantly. Figure 1 shows trends in physician return visits for patients with a diagnosis of MS, on the y-axis. These trends suggest there was a decrease in the proportion of visits involving neurologists from 1998 to 2004 and that there was a greater likelihood diagnostic/screening service provided/ordered during that period. The trend with regards to primary care providers appears to have a substantial variation from year to year.

Of the FDA-approved agents for MS, use of Avonex appeared to exhibit a downward trend (27% in 1998 to 11% in 2000), whereas that of Copaxone and Betaseron appeared to show an upward trend. However none of these trends were significant at 0.05 when using a chi-square test to detect a difference in proportions. When combined, IMA use did not show any statistically significant trend (p = 0.5113).

**Figure 2:**
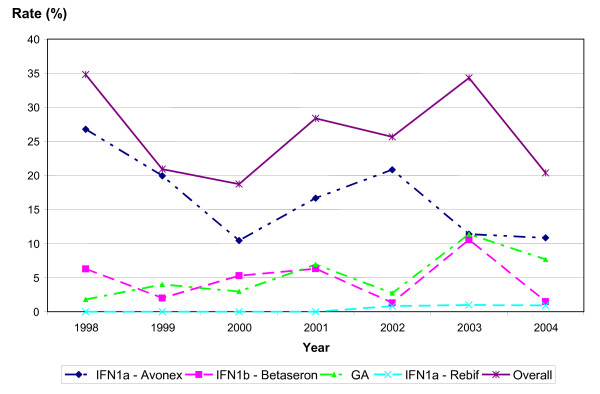
Estimated IMA prescription percentages in return visits, NAMCS data, 1998–2004.

Among the three physician groups, neurologists accounted for 78% of Avonex, and 100% of Betaseron/Copaxone prescriptions. Figure 2 shows the estimated IMA prescription percentages for return visits with a diagnosis of MS. Internists and family medicine practitioners prescribed 12% and 11% of Avonex but negligible numbers for Betaseron and Copaxone. Neurologists also prescribed IMAs more often than the other two groups combined (38% versus 9%, p = < 0.0001).

Table 2 shows logistic regression analysis predicting the probability of IMA prescription during a visit. Although the adjusted odds of IMA prescription remained equal to or smaller for all years compared to 1998, the results were not statistically significant. The odds for age group 40–59 was statistically significant when compared to the 20–30 age group (OR = 0.40). In addition, female patients were more likely to be on IMAs (OR = 1.98), and patients on Medicaid insurance were less likely to be prescribed IMAs (OR = 0.34).

## Discussion

The NAMCS data provide information about MS patients and trends in their outpatient management. NAMCS data are used by public health policy makers, health services researchers, medical schools, physician associations and epidemiologists to describe and understand the changes that occur in medical care requirements and practices. It is important to understand that data are constructed on a sample of visits rather than a sample of people. The NAMCS surveys basically provide national estimates.

Apart from obtaining basic data concerning age, gender, and geographical distribution of MS patients across the US, our goal was to evaluate the proportion of patients with an established diagnosis of MS receiving FDA-approved medications. We also studied how treatment was influenced by type of treating physician, i.e., neurologist, internist or family medicine practitioner and geographic location.

According to the national MS society, the incidence of MS in the US is about 1/1,000; we found an estimated 6.7 million MS outpatient visits occurred between 1998–2004, or 3.4 visits/1,000 patients/year. We found that women were seen four times as often as men, and Caucasians > non-Caucasians, a trend that perhaps points to increased prevalence among Caucasian women more than any one ethnic group. Not surprisingly, neurologists prescribed IMAs more often (21%) than family practitioners/internists (8%). The majority of patients seeing family practitioners or internists were on no IMA therapy. The use of Avonex declined from 1998 and could have been triggered by the perception that it has sub-optimal dose/frequency [15,16]. The fact that a greater proportion of MS visits (56%) were directed to neurologists in MSAs than in non-MSAs (26%, p < 0.0001) perhaps reflects better patient access to specialty care services in MSAs.

MS is among the leading causes of disability in young adults [17]. The natural history of MS suggests that disability accumulates over time and the use of IMAs reduces the frequency of new enhancing lesions, relapses and rate of cerebral atrophy [18-20]. The advent of Betaseron, Avonex and Copaxone represents a major breakthrough in MS therapy and three large placebo-controlled, double-blind studies have demonstrated efficacy [8-10]. Although each clinical trial had unique features and differences that make direct comparisons erroneous, published results demonstrate a clear benefit of IMAs for decreasing relapses and probability of sustained clinical disability progression in patients with MS [21]. Additionally, data from Controlled High Risk Avonex Multiple Sclerosis Study (CHAMPS; [22]) and Early Treatment of MS (ETOMS; [23]) studies suggest that initiating treatment with Avonex or Rebif early could perhaps delay the development of clinically definite multiple sclerosis. More recently, Betaseron has been shown to be of benefit in clinically isolated syndromes [24] while Rebif has been shown to be superior to Avonex in the EVIDENCE (Evidence of Interferon Dose-Response: European North American Comparative Efficacy) study [25].

The NAMCS data analysis shows that 62% of established MS patients seeing neurologists and 92% of those seeing family medicine practitioners or internists were not being treated with IMAs. The increased use of IMAs by neurologists probably reflects greater awareness of the drugs' availability and their use by specialists who more often treat patients with MS. Additionally, an association between co-payments and IMA use in patients with private-sector health plans may have a role (26). It would be important to test whether reducing co-payments for MS treatment would reduce the use of other health care services through better MS treatment that modifies the course of illness.

### Limitations in our data analyses

The reasons for the generally low IMA utilization rate were not obvious from analysis of the NAMCS data. It is plausible that some individuals with MS have mild symptoms during the initial phase of the illness, and either the patient or the physician could decide to defer treatment. Other factors for the low IMA use could be a relatively new MS therapy in the market whose risk/benefit ratio is not obviously evident, patients' lack of awareness about the pros and cons of treatment, being advised by their physician that they did not meet criteria for taking it, differences in physician communication styles, physician beliefs about the appropriateness of the drug, physician perceptions about internal organizational constraints and policies regarding its use, or some combination of these factors. Academic neurologists were not excluded from this survey – the NAMCS data are collected on individual outpatient office visits and are then weighted to reflect national estimates.

Because our data are drawn from a survey of physician office visits for MS, data drawn from tertiary care clinics or advocacy organizations may have been excluded. In addition, logistic regression analysis revealed no association between IMA prescription and type of insurance, although this was not one of our aims of our study.

The possible impact of unmeasured co-variables cannot be ignored in interpreting our findings particularly as applied to IMA use among non-neurologists. Additionally, data on longitudinal follow-up of patients are unavailable in the NAMCS databases, a significant drawback. The reason for decline in Avonex use between 1998–2000 remains unexplained and could represent sample variation. Analysis of newer data could determine how the use of IMAs is evolving.

To improve IMA prescription practices, a possible solution would be to pinpoint variations in service delivery and to initiate longitudinal follow-up studies. Above all, strategies for educating both neurologists and non-neurologists about the benefits of initiating IMA use in MS patients and in continuing their use remain critical to improving long-term patient outcomes in the treatment of MS.

## Conclusion

Strategies that lead to improved use of IMAs in the management of MS in the outpatient setting are needed. Our study found that a significant proportion of established MS patients visiting either neurologists or family medicine practitioners/internists were not using IMAs. Despite limitations inherent in our study, outpatient management of MS remains far from ideal in the USA.

## Competing interests

The author(s) declare that they have no competing interests.

## Authors' contributions

JRA was responsible for the study design, organization, analyses and manuscript preparation. FC, a bio-statistician affiliated to the Wake Forest University helped analyze the NAMCS data into meaningful tables and figures. CAO, SER and SRF contributed to the manuscript through critical analyses, proof-reading and data interpretation.

## Pre-publication history

The pre-publication history for this paper can be accessed here:



## Figures and Tables

**Table 1:** Demographics for overall and return MS visits to neurologists (N), family practitioners (FP), and internists (I)

	Overall (1998–2004)	Overall MS visit rate per year per 1,000 US residents*	Return MS visits to N, FP, and I (1998–2004)
Estimated no. of MS visits	6,682,288	3.38	4,959,782
**Specialty**			
Neurologists	50.7%	1.72	59.6%
Family practitioners	20.7%	0.70	25.6%
Internists	11.0%	0.37	14.8%
Other	17.6 %	0.60	-
Gender: females	77.6%	2.63	77.1%
Race: Caucasian	90.4%	3.06	88.9%
**Metropolitan status: (MSA)**	80.8%	2.73	76.5%
**Age group**			
0–19	2.0%	0.07	0.88%
20–39	20.8%	0.70	18.4%
40–59	57.9%	1.96	59.5%
60+	19.3%	0.65	21.2%
Primary care physician	32.3%	1.09	39.8%
**Region**			
Northeast	26.7%	0.90	25.1%
Midwest	19.4%	0.66	20.1%
South	25.1%	0.85	28.2%
West	28.9%	0.98	26.5%

* Based on 2000 census population.

**Table 2:** Logistic regression analysis predicting the probability of IMA prescription during a visit

	Odds ratio	95% Confidence intervals
2004	0.43	(0.13, 1.48)
2003	0.96	(0.35, 2.64)
2002	0.89	(0.24, 3.32)
2001	0.75	(0.23, 2.47)
2000	0.47	(0.13, 1.63)
1999	0.33	(0.09, 1.30)
1998	Reference	-
0–19	0.39	(0.06, 2.50)
20–39	Reference	-
40–59	0.40	(0.21, 0.79)
60+	0.42	(0.15, 1.15)
Female	1.98	(1.09, 3.58)
Neurologist	6.69	(2.87, 15.59)
Caucasian	0.62	(0.31, 1.22)
Private insurance	Reference	-
Medicare insurance	0.86	(0.43, 1.72)
Medicaid insurance	0.34	(0.11, 0.99)
Other insurance	1.26	(0.47, 3.38)
